# A Clinical Study of the End Results of Some Focal Infections*Read before the Section on Practice of Medicine at the Seventy-first Annual Session of the American Medical Association, New Orleans. April. 1920. Reprinted from *The Journal N. D. A.*, June 12, 1920.

**Published:** 1920-07

**Authors:** Bryce W. Fontaine

**Affiliations:** Memphis, Tenn.


### A CLINICAL STUDY OF THE END RESULTS OF SOME FOCAL INFECTIONS *

BY BRYCE W. FONTAINE, M.D., MEMPHIS, TENN.

In a casual survey of the literature on focal infections, one is impressed with the fact that only recently has the subject been studied from the standpoint of its bearing on systemic disease.

Until the last decade there were few notable contributions. In 1819, Rush reported the cure of a case of rheumatism by the extraction of a tooth. William Hunter, in 1899, recorded his studies of what he termed "oral sepsis." Later, the dentists Price, Broomell and Chayes in their articles cautioned against careless dentistry and neglected oral sepsis, suggesting the probability of systemic disease ensuing. Goadby's article in 1912 claimed the direct relationship of some joint conditions to focal infection.

It remained, however, for Frank Billings and his coworkers, especially Rosenow and Davis, to bring the subject prominently before the medical profession. Too much can not be said in praise of their work, and the credit for the present knowledge of the subject must be given to them. Since the first paper by Billings appeared in 1912, tremendous enthusiasm has been manifested in this subject, many articles and one monograph appearing within the short space of eight years.

Despite this splendid work, there exists in many sources some doubt as to the part focal infections play in the production of diseases, and many are questioning whether or not any real relationship exists. A warning is sounded lest we, in our zealous efforts to eradicate sus-

---

* Read before the Section on Practice of Medicine at the Seventy-first Annual Session of the American Medical Association, New Orleans, April, 1920. Reprinted from *The Journal N. D. A.*, June 12, 1920.

pected foci, sacrifice useful organs, such as the teeth and tonsils. I realize that in many instances the pendulum has swung too far, and that some practitioners are likely to remove sound teeth or tonsils without justification. If the cases are carefully studied from every angle, and all other possible etiologic factors eliminated, the prompt relief obtained by radical measures should convince the most skeptical that it is not due to accident, coincidence, or to any effect on the mind.

In this confused state of evidence, it may be of interest to hear the clinical experience of one observer, and note the end results obtained in approximately 100 cases selected from a private and consultation practice, during a period of two years.

The relation of the focus of infection to the symptoms in question has depended on: (1) the absence of any other demonstrable cause for the symptom; (2) the failure to cure the symptom by all other methods of treatment; and (3) prompt and continued relief, with no return of the symptom, on the cure or eradication of the focus of infection.

In this study, conclusions have been arrived at largely through the co-operation of a group of nose, throat and dental specialists. Great reliance has been placed on roentgenograms of the diseased structures.

### SOURCES OF INFECTION

The teeth have been the most common source of infection, and all varieties of dental conditions, from pyorrhea to unerupted twenty-one-year molars, have been encountered. The most common lesions were either apical abscesses or granulomas at one or more roots of devitalized teeth. The diagnosis was confirmed by plates of the whole upper and lower jaws, including all devitalized teeth. Not only should infected teeth be removed, but the cavities left should be thoroughly curetted.

The tonsils were next in importance as a seat of infection. The small fibrous or submerged tonsils were quite as frequently involved as those visibly infected and greatly hypertrophied. Complete enucleation with the capsule was usually necessary for permanent relief.

The sinuses were least in importance in this series. The maxillary sinus was most frequently implicated, because of its close proximity to the throat and nose. Transillumination and roentgenograms were employed to reveal existing trouble.

There has been no experience with foci of infection in other parts of the body. Billings, however, reports them in almost every conceivable location.

### SYMPTOMS FOR WHICH RELIEF WAS SOUGHT

Pain occurring in 42 per cent. of the cases was the symptom for which relief was most frequently sought, the location usually being in the muscles of the neck, back, chest or limbs. In a few cases it occurred along the course of some nerve, frequently branches of the brachial plexus or the sciatic nerve. Its character varied from ordinary discomfort to intense agony, requiring morphine for alleviation. The painful area was not affected by changes in temperature, nor was it often associated with tenderness. It was not accompanied by fatigue or other neurotic symptoms one might expect in neurasthenia. Conditions about the teeth most frequently produced these symptoms, though some cases were due to diseased tonsils or sinuses.

Case 1.—E. A. S., a white man, aged 50, had constant pains in the right back, side and hypochondriac region, at times severe enough to require morphine. A diagnosis of gallstones had been made, and operation advised. A physical examination of the chest and abdomen was negative. The gastric contents, urine and blood were normal. The Wassermann reaction of the blood serum was negative. Only temporary relief was obtained by the usual methods of treatment. Examination of the teeth disclosed two apical abscesses. Removal of the teeth afforded immediate relief, with no recurrence of pain.

Case 2.—H. C. N., a white man, aged 35, had pain in both shoulders, causing discomfort, rather than acute suffering. It was not especially related to the joints or nerves, but affected the muscles about both shoulders. There was no tenderness. It was not affected by changes in weather. Physical examination of the heart, lungs and abdomen was negative. The urine and blood were normal. The Wassermann reaction of the blood serum was negative. Examination of the throat revealed infected tonsils. The patient was always relieved for weeks at a time, by massage and squeezing out of crypts by a throat specialist. Ordinary methods afforded no relief. Tonsillectomy was finally performed, giving immediate relief, with no recurrence of pain. The patient made a gain of 12 pounds.

Fever, occurring in 18 per cent. of the cases, was second in importance as a symptom. The fever, seldom over 101 F., was not often accompanied by chills, though in a few cases night sweats occurred. These cases were usually ambulatory, resembling, and most frequently confused with, the fever of incipient tuberculosis. The foci were about equally distributed between the teeth and the tonsils.

Case 3.—H. R. W., a white man, aged 44, had had fever for four weeks, with some malaise, an occasional night sweat and a slight loss of weight. His highest temperature was 99.8 or 100, usually in the afternoon. The patient was not ill enough to go to bed. He had been at his office almost every day. Physical examination of heart, lungs and abdomen was negative. The differential blood count was normal. There were no plasmodia. On three occasions the Widal test was negative with typhoid and paratyphoid bacilli. The Wassermann reaction of the blood serum was negative. There was slight tenderness about the first lower molar on the right side, which was devitalized and crowned a few years ago. A roentgenogram revealed an apical abscess. Removal of the tooth was followed by prompt and permanent subsidence of temperature, with a gain in weight to normal.

Chronic headache as a result of focal infection has long been suspected by throat and oral surgeons. Often in their experience the removal of infected tonsils has relieved the patient of headache of long standing. In this series, chronic headache has occurred in 4 per cent. of the cases, each depending on tonsillar infection. In every case a prompt and permanent cure has followed tonsillectomy.

Case 4.—Mrs. W. V., aged 28, white, complained of headache, which had occurred rather frequently for the last two years. It did not occur at any regular time, nor was it related to the menstrual period. The patient had had no previous illness. She had had many examinations, with negative findings and no benefit. The last physician who made an examination suggested glasses, but they caused no improvement. Physical examination of heart, lungs, abdomen and pelvis was negative. Neurologic examination was also negative. The urine, blood and gastric contents were normal. The Wassermann reaction of the blood serum was negative. An oral surgeon reported that all sinuses were negative, but the tonsils were hypertrophied, showing evidences of infection in the crypts. Tonsillectomy gave prompt relief.

General debility with loss of weight and strength, resulting from focal infection, has occurred in about 10 per cent. of the cases.

This syndrome, as a rule, occurred in middle-aged adults, without any other apparent basis, and in most instances was accompanied by obvious diseases of the teeth. No cases have occurred with infection elsewhere. In many cases it was necessary to remove all the teeth in order to get rid of the severe oral infection, all ordinary methods of treatment having utterly failed. Removal of the teeth and treatment of the gums, when promptly and effectively done, were followed by marked improvement in the general condition, with a gain in weight and strength.

Severe secondary anemia occurred in 4 per cent. of the cases. In no case, despite the ordinary methods of treatment, such as rest, feeding and the continued use of iron and arsenic, was there any perceptible improvement until the removal of the focus of infection. The cases were equally distributed between the tonsils and the teeth. In no instance did the blood present the morphologic appearance of pernicious anemia. There was usually a moderate reduction of the hemoglobin and in the number of erythrocytes, with normal differential and leukocyte counts.

Case 5.—Mrs. N. O. E., aged 26, white, complained of weakness and indigestion, beginning more than a year before. Physical examination of the heart, lungs, abdomen and pelvis was negative. The Wassermann reaction of the blood serum was negative. The urine was negative. The gastric contents after an Ewald test meal showed free hydrochloric acid 5, total acidity 16. Blood examination revealed: leukocytes, 7,300; erythrocytes, 3,800,500; hemoglobin, 65 per cent. Differential count revealed: polymorphonuclears, 63 per cent.; transitionals, 1; lymphocytes, 29; large mononuclears, 5; eosinophils, 2. Red cells were slightly irregular in size. No normoblasts were seen. There were no plasmodia. Examination of the throat by an oral surgeon showed the tonsils to be hypertrophied and infected. Tonsillectomy resulted in an almost immediate gain of 12 pounds. Four months later the blood was normal.

The relationship of focal infections to albuminuria and nephritis has been proved and referred to by several observers, prominent among whom are Ophuls, Klotz, LeCount and Jackson. In this series there were six cases apparently dependent on this cause. In a majority the focus of infection was in the tonsils, though in two it was about the teeth. In every instance, after tonsillectomy or removal of the oral infection, there was a progressive improvement, with a disappearance of albumin and casts from the urine.

Case 6.—Mrs. W. L. S., aged 32, white, complained of aching, loss of weight, and pain in the back and shoulders. Physical examination of the heart, lungs and abdomen was negative. The blood was negative. The systolic blood pressure was 110, the diastolic, 80. The urine showed a moderate trace of albumin, with numerous hyaline and granular casts. After a Mosenthal test meal for renal function, the urine showed a total output of 1,240 c.c., a normal variation in the specific gravity of day specimens. The specific gravity was inversely proportional to the volume of the specimens. There was diuresis after the first two meals; after the third meal, it was very slight. The night urine was small in volume, with high specific gravity. There was adequate salt elimination, and adequate nitrogen concentration in the night specimen. There was moderate fluid retention, and slight nitrogen retention. The salt concentration was low. The phenolsulphonephthalein output at the end of two hours was 30. The tonsils were small and fibrous, showing pus in the crypts. Treatment by dieting and rest for some months resulted in no improvement. Tonsillectomy eight months ago gave immediate relief from pain, with a gain of 20 pounds. The urine three weeks ago was absolutely normal.

The association of achylia gastrica with focal infections has been referred to by several observers, cases being reported by Beck and others. Every gastroenterologist has witnessed the improvement in many cases of this character, following the cure of oral sepsis. In this series there were six cases, all proved by the fractional method of analysis to be true achylias. In each there seemed to be considerable infection in the mouth, but in no other location. In some of the cases the extent of the infection necessitated the extraction of all the teeth. Without exception, improvement followed the cure of the oral sepsis. Free hydrochloric acid reappeared to some extent in every case, and in a few it finally reached the normal level.

Case 7.—Mrs. E. T., aged 38, white, complained of loss of weight and strength, discomfort in the abdomen, gas and constipation. Physical examination of the heart, lungs and abdomen was negative. The blood and urine were negative. The Wassermann reaction of the blood serum was negative. The gastric contents by fractional analysis showed a total acidity of 12 at the end of one and one-half hours. There was no free hydrochloric acid throughout the test, which was carried on for two and one-half hours. There was no occult blood or lactic acid. Roentgenographic examination of the stomach, after a barium meal, was negative, except for hypermotility, the stomach being empty at the end of three and one-half hours. The patient had previously had several attacks of tonsillitis. The tonsils were recognized to be hypertrophied and infected. Under dieting and free hydrochloric acid, along with a modified rest cure, there was only slight improvement. Tonsillectomy was performed eight months ago, with immediate improvement in the patient's general condition, with a gain in weight and strength, and improvement in stomach symptoms. The last analysis of gastric contents, one month ago, showed free hydrochloric acid 12, and a total acidity of 20.

Vascular hypertension of moderate degree, indicated by a systolic pressure of from 150 to 170, occurred in 6 per cent. of the patients, all middle-aged persons, principally women. In these cases the condition was apparently not due to nephritis, because of the negative urine findings. There may, however, have been some chance of error, if we consider as a clinical entity the hypertension of the climacteric so well described by Reisman, and probably due to some endocrine dysfunction. In every case the hypertension seemed to resist all ordinary therapeutic measures, such as dieting, rest, and vasodilators. Without exception, the infection was about the teeth, and on its cure there was a prompt reduction in the blood pressure, though it rarely reached normal.

While the report of the foregoing cases does not comprise all in which there has been a direct relationship or dependence on focal infection, others have occurred with rarity, and time forbids mentioning them.

Finally, a word must be said about joint conditions. No acute joint cases are tabulated in this series. There have been perhaps eight cases of chronic arthritis, usually of the type of arthritis deformans, with definite deformity and disability of many joints. An unsuccessful attempt was made to benefit these patients by curing foci of infection existing in the teeth, tonsils or sinuses.

### CONCLUSION

While this report is necessarily brief, I hope it is sufficiently convincing to prove that often such conditions as pain, fever and headache are directly traceable to focal infections, and that the most effective measure for relief is complete eradication or a cure. The diagnosis of focal infection is made reluctantly, not until every other means of arriving at the cause in these cases has been exhausted, and then only with the co-operation of other men, who are conservative and especially skilled in their several lines. These end results have been carried over two years, and during this time there has not been a return in one of the cases reported.

### ABSTRACT OF DISCUSSION

Dr. Frank Billings, Chicago: Focal infection as a cause of disease has come to stay. But, like every other principle in medicine, it has its limitations. Many of those who accept it have not thought of just what the term means. A focus of infection differs from a focal infection. A focus of infection may give rise to focal infection, or it may give rise to intoxication of the body. What is meant by infection? Infection means the invasion of the body by micro-organisms that have the power of reproduction in the host, of producing reactions within the tissues of the host, and the reaction producing abnormal phenomena, which we term clinical manifestations of disease. Focal infection means the invasion of the body from a focus of pathogenic organisms, and these organisms have the power of reproduction or of multiplication within the host. Much of the fallacy and failure in the treatment or management of the patients who suffer from what may be proved to be focal infection is due to the fact that the surgeon or physician in charge removes a focus, which may be the right one, and then neglects any further management of his patient. And, if what I have just said is true, then the removal of the focus has not disturbed the organisms already in the tissues of the body. If you have removed the true focus, you have simply prevented the invasion of the tissues by additional organisms from the primary focus. Focal infection is usually an invasion by pathogenic agents of the tissues through the blood stream. They lodge in the tissues, they produce reactions, dependent on their character and on their virulence. They remain there if the defenses of the host are not sufficient to kill them or at least to drive them from the body. And unless, in the treatment of chronic infectious arthritis, for instance, that fact is borne in mind, the removal of the focus will usually not greatly benefit the patient, or at any rate not the majority of patients. Therefore, what is your duty? Our duty is to build up the resistance of the host against the invaders already there by all of the known means of support of the patient from the beginning of treatment. Can we use anything specific? The use of antigens in the form of dead bacteria, injected subcutaneously, in my experience, is not justified. I have used them all, controlling the work with all the knowledge we have, using the autogenous, the nonautogenous, the activated and the nonactivated. The controls got along just as well as those that had the vaccines given them carefully. May one use some other specific? It is not uncommon now to read that a foreign protein antigen, injected introvenously, will restore the protective powers of the body, and drive out the pathogenic invaders. Apparently, many men have had success. But immunologists are not able to tell us much, if anything, of the modus operandi. It produces serious reactions, and we are using it in a haphazard way. The conscientious man is taking the health, and possibly the life, of the individual in his hands when he uses these substances. I do not say, do not use them, but I believe that we should approach that part of the treatment with judgment and discretion.

Dr. Joseph H. Pratt, Boston: The subject of focal infections in its present-day conception is very complex. There is much need of careful clinical study. Dr. Fontaine took a conservative position. A defect in most of the papers on this subject is that they give only the immediate results of removing foci of infection, but Dr. Fontaine has followed his cases for from one to two years, and the results are striking. I wish every physician in my section of the country could have heard the words just spoken by Dr. Billings, because the idea of focal infection, especially abscesses about the teeth, as a cause of chronic disease has spread like wildfire. At the present time many physicians, if they have an obscure case, simply advise the patient to go to a dentist and have the dead teeth removed. When this is done the patients are often turned adrift. Even living teeth may be extracted. The treatment in cases of psychoneuroses may consist simply in the extraction of teeth or of a tonsillectomy. Last summer I saw a woman with a mild neurasthenia, without a symptom of chronic infection, who was sent by her physician to an oral surgeon, who, according to the patient's statement, was the leading authority in the state. Fifteen teeth were extracted. I saw the roentgenograms later and only eight of the fourteen teeth were dead! Her symptoms became more marked after the teeth were removed. Many of the chronic cases that drift from physician to physician are patients suffering from psychoneuroses, and while it is proper, of course, to treat the teeth if they are in bad condition, it is a mistake to consider that in these cases the discovery and removal of infected teeth constitute proper diagnosis and treatment. If an infection has become generalized, removal of the local focus will not result in cure. In a rare case of recurrent thrombophlebitis under my observation for several years there was chronic antral suppuration. Treatment of the empyema was followed by improvement for about one year; then new thrombi formed. Careful examination has revealed no focus of infection outside of the inflamed veins, but every few months he has an attack of phlebitis with fever. Support from pathologists and immunologists is needed in solving the clinical problems connected with focal infection.

Dr. Frank B. Wynn, Indianapolis: In the olden days men were very prone in obscure cases to hide behind the liver; and a little later they found recourse in malaria to cover their sins of ignorance; and I wish to join with Dr. Pratt in emphasizing still further the point he made, namely, that the sin which we are very apt to commit at this time in regard to this matter of focal infection is that in cases with obscure symptoms we will hastily put the blame on the tonsils or on the teeth, without due clinical investigation of that case. It behooves us not to be content with looking at the tonsils or the teeth, but to search the body for other foci of infection. One other point is there which I wish to make, namely, that, granting the importance of a focus of infection, from which there is fed into the system the infectious material that gives rise in one case to rheumatic symptoms, in another to neuritis or to a neurosis, let us beware of the manner in which we proceed to correct that focus of infection. A patient had pain in the back, with tenderness, which was designated by her physician as lumbago. She unquestionably had bad teeth. On the advice of her physician they were extracted. In twenty-four hours following the removal of her teeth, she had severe pain throughout the whole extent of her spinal column, which rapidly developed into a typical spondylitis deformans. Undoubtedly, many of you have discovered bad tonsils, associated with the development of a chronic nephritis. You have removed the tonsils as the focus of infection, only to find that following the operation there has been an extreme aggravation of the symptoms, and in a few cases very disastrous results.

Dr. W. S. Thayer, Baltimore: In dealing with many obscure conditions, looking for and properly treating a focus of infection, which may be a contributory cause of the trouble, we sometimes forget that our responsibility goes considerably beyond the point of finding a focus which may possibly be the seat from which the infection which has caused the trouble has come, and of considering its proper treatment. The attempt to treat these foci of infection properly has made common surgical operations which were not so common years ago; and it has brought into use certain specialties of surgical procedure which were not common years ago, surgical procedure of a very delicate character, which only the best trained man is capable of carrying out. I know many men whom I would be glad to have take out my appendix, if it were necessary. I know very few men who I would be willing to have take out an adherent tonsil or interfere with ethmoid disease. The proper treatment of ethmoid disease and of gravely infected tonsils is often a serious matter. Those are very delicate procedures, which should only be done by very well-trained surgeons. And there are relatively very few well-trained surgeons in these special branches. It is our duty to consider very carefully to whom our patients are sent; and, furthermore, to follow them up afterward. The common treatment of the teeth in individuals whose trouble is supposed to depend on infection often is barbarous in the extreme. The reckless manner in which teeth are extracted today is a scandal to the medical profession. The very commonest infection in the mouth is probably gingivitis or pyorrhea alveolaris, which is not properly treated in the vast majority of cases. Very few dentists pay proper attention to pyorrhea alveolaris, and it is the duty of the physician to find one who does. I often do not know what to do when I have to refer a patient to someone for the treatment of such a condition. Dead teeth are opened and extracted on suspicion, simply because they are dead. Such a procedure is shameful. The extraction of a dead tooth, because it is dead, is unpardonable.

Often apical necroses are found in teeth, and those teeth are instantly extracted. In many instances that is an entirely wrong procedure. An apical necrosis does not at the moment often mean a very grave infection. Very often it is easy to treat the condition without extracting the teeth. A sufficiently free opening, with curetting, and amputation of the root, done by a skilful man, will save many teeth which are today sacrificed recklessly. When we send a patient to a dentist to have his teeth extracted, it is not only necessary to see that he is properly cured, but it is unfortunately necessary very often to watch the patient to see that he himself treats the cavities properly. I know dentists of the highest reputation in their communities who extract a tooth, curette well the socket and then dismiss the patient; and I have on more than one occasion met such a patient several years later, and found that he had suppurating cavities, the necrosis still continuing, well closed in, simply because the cavity had not been properly treated.

